# Global inequitable distribution of seminal studies of chemotherapy-induced nausea and vomiting

**DOI:** 10.1007/s00520-026-11103-0

**Published:** 2026-08-15

**Authors:** Gregory W. Chai, Yigeng Tan, Jennifer Leigh, Daichi Watanabe, Hirotoshi Iihara, Ronald Chow

**Affiliations:** 1 Temerty Faculty of Medicine, University of Toronto, Toronto, ON Canada; 2 Odette Cancer Centre, Sunnybrook Health Sciences Centre, Toronto, ON Canada; 3 Practical Pharmaceutical Research Center, Gifu Pharmaceutical University, 1-25-4 Daigaku-Nishi, Gifu, 501-1196 Japan; 4 Department of Pharmacy, Gifu University Hospital, 1-1 Yanagido, Gifu, 501-1194 Japan; 5 Cancer Center, Gifu University Hospital, 1-1 Yanagido, Gifu, 501-1194 Japan; 6 Centre for Evidence-Based Medicine, University of Oxford, Oxford, UK

**Keywords:** Chemotherapy-induced nausea and vomiting, Cancer therapy, Antiemetic therapy

## Abstract

**Background:**

Chemotherapy-induced nausea and vomiting (CINV) remains one of the most quality of life limiting toxicities of cancer therapy. Decades of trials have produced robust antiemetic evidence, but their applicability to resource-limited settings where guideline-recommended regimens are frequently inaccessible has not been characterized.

**Methods:**

We reviewed the reference lists of the 2023 MASCC/ESMO, 2025 NCCN, and 2020 ASCO antiemetic guidelines to identify all primary research articles cited as evidence for antiemetic therapy. Review articles were excluded. For each study, we extracted country of origin, income classification, age, sex distribution, primary cancer type, chemotherapy regimen, chemotherapy emetogenicity, and antiemetic regimen. Findings were synthesized descriptively.

**Results:**

From the reference list, 213 articles were identified. Two could not be retrieved and one was identified as a review, leaving 210 studies for extraction. When the same patient population was reported across multiple studies, the reports were consolidated and represented by the most comprehensive study, resulting in 195 unique populations for analysis. Most studies enrolled patients receiving highly emetogenic chemotherapy (63.1%), followed by moderately emetogenic (27.2%), minimally emetogenic (8.7%), and low emetogenic (1.0%) regimens. The evidence base was overwhelmingly derived from high-income countries (HICs): Of 191 studies with a determinate income classification, 82.7% originated in HICs and only 17.3% in low- and middle-income countries, persisting across every emetogenicity category.

**Conclusions:**

The evidence supporting contemporary CINV guidelines is concentrated in high-income settings. As alternative, lower-cost antiemetic regimens for resource-limited settings are derived from this same evidence base, further studies are needed to assess their effectiveness in these settings.

**Supplementary information:**

The online version contains supplementary material available at https://doi.org/10.1007/s00520-026-11103-0.

## Introduction

Chemotherapy-induced nausea and vomiting (CINV) is among the most common adverse effects of anticancer systemic chemotherapy, affecting most patients on emetogenic chemotherapy without prophylaxis [1]. It is categorized by temporal pattern (acute, delayed, long-delayed) and emetogenic risk: high (HEC), moderate (MEC), low (LEC), and minimal [1]. Beyond acute discomfort, it impairs quality of life, causes dehydration and malnutrition, and strains healthcare resources [2]. This drives nonadherence as patients with severe early-cycle symptoms more often delay, reduce, or abandon potentially curative treatment [3].

Much of the supportive care research in the past four decades has been devoted to antiemetic prophylaxis. The serotonin (5-HT3) receptor antagonists [4], neurokinin-1 (NK1) receptor antagonists [5], corticosteroids [6], and olanzapine [7] now form the backbone of evidence-based prophylaxis. This accumulated evidence has led to widely adopted clinical practice guidelines, including those of the Multinational Association of Supportive Care in Cancer and the European Society for Medical Oncology (MASCC/ESMO) [8], the National Comprehensive Cancer Network (NCCN) [9], and the American Society of Clinical Oncology (ASCO) [10].

Despite all this research, there may be inadequate access to antiemetics in resource-limited settings [11]. In recognition of this, MASCC has issued dedicated consensus recommendations for resource-limited settings, proposing alternative, lower-cost regimens intended to approximate the efficacy of the standard of care when the preferred regimen cannot be accessed [11].

However, the representativeness of that underlying evidence for this guideline has received little attention. If the trials supporting current guidelines were conducted predominantly in high-income countries (HICs), extrapolating their findings to resource-limited populations, who may differ in supportive care infrastructure, organizational factors, and concomitant medication availability, warrants caution [12]. Thus, the aim of this study is to characterize the geographic origin and income-setting distribution of the primary studies cited as evidence across the contemporary MASCC/ESMO, NCCN, and ASCO antiemetic guidelines, reporting this distribution both overall and by chemotherapy, in order to determine whether low- and middle-income countries (LMICs) are underrepresented.

## Methods

We examined the reference lists of three contemporary antiemetic guidelines as of April 1, 2026, consisting of the 2023 MASCC/ESMO consensus recommendations [8], the 2025 NCCN antiemesis guideline [9], and the 2020 ASCO antiemetic guideline [10] to identify all primary research articles cited as evidence for antiemetic therapy. All primary research articles were eligible for inclusion while review articles were excluded as their constituent primary studies were captured separately. When the same underlying patient population was reported across multiple publications, these reports were consolidated and represented by the most comprehensive publication to avoid double counting. The most comprehensive study was used to extract all population-level characteristics, including country, income classification, enrollment year, demographic characteristics, primary cancer type, index regimen, and emetogenicity. Variables not reported in the most comprehensive study were supplemented from other linked reports when the information was directly generalizable to the same study population, such as when population size and characteristics were consistent across reports.

For each eligible study, two reviewers (GWC, MT) extracted the following variables on a standard extraction template consisting of the following variables: country of origin, year, LMIC or HIC, age—median (IQR or range), age—mean ± SD, percentage female, primary cancer type, chemotherapy regimen, chemotherapy emetogenicity, and antiemetic regimen (5HT3, NK1, DEX, OLN, other). Chemotherapy emetogenicity was classified into high (HEC), moderate (MEC), low (LEC), and minimal emetic risk categories according to the 2023 MASCC/ESMO emetic risk classification of antineoplastic agents [13]. If a study enrolled a mixed population, emetogenicity was assigned on the basis of the predominant or index regimen as described by the authors.

To reflect the economic context in which the research was actually conducted, we classified LMIC or HIC based on the World Bank classification at the time of the patient’s first enrollment if that information was available. Otherwise, they were classified based on the time of publication. Multinational trials that did not specify the contributing countries or spanned multiple income groups were classified as HIC based on the assumption that conducting a trial across several countries requires a level of funding, regulatory capacity, and research infrastructure that is more likely to be found in wealthier health systems. This approach was necessary because participating countries were not consistently reported across studies, preventing uniform country-level classification. Notably, all multinational trials with identifiable participating countries included at least one high-income country.

Given the heterogeneity of the included studies in design, population, and outcome, we did not conduct a meta-analysis and findings were synthesized descriptively. The full study-level extraction is provided in Supplemental Table 1.

## Results

### Study characteristics

The search of guideline reference lists found 213 studies. Two articles were excluded because their full texts could not be retrieved, and one review article was excluded as it did not meet the inclusion criteria for primary studies, leading to 210 primary studies. Of these, 15 studies were studies reporting on the same underlying patient population as another included study and they were consolidated with the most comprehensive study. After this consolidation, the 210 studies corresponded to 195 unique study populations, which formed the basis for all analyses [14–223].

Studies started enrollment between 1978 and 2022. Among the 147 studies with an extractable year of enrollment, the number of trials rose drastically over time: one study (0.7%) between 1970 and 1979, 11 studies (7.5%) between 1980 and 1989, nine studies (6.1%) between 1990 and 1999, 28 studies (19.0%) between 2000 and 2009, 93 studies (63.3%) between 2010 and 2019, and five studies (3.4%) between 2020 and 2022 (Table 1). Age reporting was inconsistent, where 112 studies (57.4%) reported a median, IQR, or range, 110 studies (56.4%) a mean, and 5 studies (2.6%) reporting neither. Ages generally fell in the fifth and sixth decades. The proportion of female participants was reported in 192 studies (98.5%), with a median of 47.1% and a full 0–100% range reflecting sex-specific malignancies. Antiemetic regimens reflected the era studied where 5-HT3 receptor antagonists appeared in most studies, NK1 antagonists in a substantial share of more recent trials, and olanzapine in a smaller but growing number.

**Table 1:** General study characteristics (*n* = 195)

Characteristic	Value
Total number of included primary studies	195
Publication year, range	1978–2022
Studies with a reported start of enrollment, *n*	147
1970–1979	1 (0.7%)
1980–1989	11 (7.5%)
1990–1999	9 (6.1%)
2000–2009	28 (19.0%)
2010–2019	93 (63.3%)
2020–2022	5 (3.4%)
Age reported as median, range, or IQR, *n* (% reported)	112 (57.4%)
Age reported as mean, *n* (% reported)	110 (56.4%)
Age not reported at all, *n* (% not reported)	5 (2.6%)
Sex reported, *n* (%)	192 (98.5%)
Median female proportion (range)	47.1% (0–100%)
Chemotherapy emetogenicity, *n* (%)	
HEC	123 (63.1%)
MEC	53 (27.2%)
Minimally emetogenic	17 (8.7%)
LEC	2 (1.0%)
Antiemetic drug class, *n* of studies (%)	
5-HT3 receptor antagonist	176 (90.3%)
NK1 receptor antagonist	107 (54.9%)
Dexamethasone	156 (80.0%)
Olanzapine	45 (23.1%)

Percentages for age and sex use all 195 studies as the denominator; year subcategories use the 147 studies with an extractable publication year. Antiemetic drug class counts are nonexclusive, as most studies evaluated combination regimens

*HEC* highly emetogenic chemotherapy, *MEC* moderately emetogenic chemotherapy, *LEC* low emetogenic chemotherapy

### Distribution by chemotherapy emetogenicity

The evidence is concentrated on the most emetogenic regimens where 123 studies (63.1%) involved HEC regimens and 53 studies (27.2%) MEC regimens versus 17 studies (8.7%) minimally emetogenic and only 2 studies (1.0%) utilizing LEC regimens (Table 1).

### Distribution by income setting

Income setting could be identified for 191 of 195 studies (four studies had indeterminate income settings) (Table 2). Of these, 158 (82.7%) were conducted in HICs and 33 (17.3%) LMICs. The HIC evidence itself was concentrated where the USA conducted 43 studies and Japan in 37 studies supplied just over half of all HIC studies (80 of 158 studies, 50.6%), distantly followed by Italy in 15 studies and Korea in 7 studies. Multinational trials that did not specify the contributing countries or spanned multiple income groups comprised 40 HIC-classified studies. The entire LMICs came from just seven countries and were dominated by China in 15 studies and India in nine studies, together composing 72.7% of LMIC studies, with the rest from Thailand (*n* = 4), Iran (*n* = 2), and one each from Egypt, Turkey, and Pakistan. No study was conducted solely within an African or Latin American country, and these regions appeared only within multinational collaborations.

**Table 2:** Country of origin of included studies

Income setting/country	Studies, *n* (%)
HIC	158
USA	43 (27.2%)
Japan	37 (23.4%)
Italy	15 (9.5%)
Korea	7 (4.4%)
Australia	3 (1.9%)
France	2 (1.3%)
Canada	2 (1.3%)
Netherlands	2 (1.3%)
Germany	2 (1.3%)
Other single HIC country^*^	5 (3.2%)
Multinational	40 (25.3%)
LMIC	33
China	15 (45.5%)
India	9 (27.3%)
Thailand	4 (12.1%)
Iran	2 (6.1%)
Egypt	1 (3.0%)
Turkey	1 (3.0%)
Pakistan	1 (3.0%)
Indeterminate	4

Percentages were calculated by how many HIC and LMIC were in each respective category

*HIC* high-income country, *LMIC* low- and middle-income country

^*^Other single HIC countries (one study each): Spain, Denmark, Finland, Belgium, and Hong Kong. Multinational trials were classified as HIC

This income imbalance was present across every emetogenicity level (Table 3). For each HEC, 96 of 119 (80.7%) were HIC and 23 (19.3%) LMIC; for MEC, 45 of 53 (84.9%) HIC and 8 (15.1%) LMIC; for minimally emetogenic chemotherapy, 16 of 17 (94.1%) HIC and 1 (5.9%) LMIC. The two LEC studies comprised one HIC and one LMIC study.

**Table 3:** Distribution of studies by income setting and chemotherapy emetogenicity

Emetogenicity	HIC, *n* (%)	LMIC, *n* (%)	Indeterminate, *n*	Classifiable total number of studies
HEC	96 (80.7)	23 (19.3)	4	119
MEC	45 (84.9)	8 (15.1)	0	53
Minimally emetogenic	16 (94.1)	1 (5.9)	0	17
LEC	1 (50.0)	1 (50.0)	0	2
Overall	158 (82.7)	33 (17.3)	4	191

Percentages are calculated using the classifiable total (HIC + LMIC) within each row as the denominator; indeterminate studies are excluded from percentages

*HIC* high-income country, *LMIC* low- and middle-income country, *HEC* highly emetogenic chemotherapy, *MEC* moderately emetogenic chemotherapy, *LEC* low emetogenic chemotherapy

## Discussion

Our review of the primary studies cited by three contemporary antiemetic guidelines demonstrated that the evidence base informing CINV prophylaxis recommendations is overwhelmingly derived from high-income countries. Of 191 studies with a determinate income classification, 158 (82.7%) were conducted in HICs and only 33 (17.3%) in LMICs, with the USA and Japan alone accounting for just over half of all high-income studies and just seven middle-income countries, led by China and India, contributing to the entirety of LMICs.

This notable imbalance was persistent across the evidence base, across every level of chemotherapy emetogenicity, from highly emetogenic regimens (19.3% LMIC) through moderately (15.1%) and minimally emetogenic regimens (5.9%). The evidence base was also concentrated temporally where nearly two-thirds of datable studies started enrollment in the 2010–2019 time period.

These findings support previous studies suggesting that clinical trial evidence is geographically concentrated. Analyses of registered trials and oncology guideline literature have repeatedly shown that most cancer research is conducted in high-income settings [224]. Although the global cancer burden is increasingly localized in LMICs, the trials that established current standards for antiemetic care were conducted almost exclusively in higher-income settings [225, 226].

The pattern is particularly evident for CINV as the guidelines built on this evidence acknowledge the problems in the access of supportive care, issuing recommendations for resource-limited settings where the most effective agents such as NK1 receptor antagonists are often unavailable or unaffordable [11]. Our analysis adds an additional concern in the generalization to LMICs. The recommended antiemetics are less accessible in LMICs, and simultaneously, the evidence supporting their effectiveness and the data informing lower-cost alternatives have been generated primarily in populations and healthcare systems that differ materially. Antiemetic response is influenced by factors such as pharmacogenomic variation, dietary counseling, and care infrastructure, which may differ across populations [227–229]. While this does not imply that current recommendations are inappropriate for LMIC patients, their effectiveness in these settings should be further investigated.

This study builds on previous literature where earlier work has compared outcomes between different regional cohorts [230], mapped the geography of oncology trials in general [224], or the designs and outputs of LMIC versus HIC [226]. However, our focus is distinct as we evaluate the landmark clinical trials that have shaped the antiemetic oncology evidence base. This is important as guidelines translate trial evidence into global standards of care and may not be representative in settings that have contributed minimal data to the underlying evidence base. As a result, the applicability of guideline recommendations may vary across populations. In the context of CINV care, the patients who present more of a challenge in antiemetic regimen selection are also frequently the ones underrepresented in clinical trials, making the available evidence less directly relevant to their care.

These findings support the need for broader geographic representation of supportive care research. Lower-cost study designs, such as registry embedded studies, adaptive platform trials, and comparative effectiveness evaluations of antiemetic regimens available in LMICs can generate evidence that is directly applicable to the settings in which it will be used. Research is needed to determine the best antiemetic regimens for healthcare settings where medication options are limited so that treatment recommendations can be based on local evidence rather than adaptations of regimens developed in HICs.

This study has several limitations. The analysis is dependent on the accuracy and completeness of the underlying publications. Country and income classifications were assigned from published reports, and several multinational studies did not specify contributing countries and classifying these trials as high income may have introduced some misclassification, potentially underestimating LMIC participation. Income level also could not be determined for four studies. In addition, some study characteristics were inconsistently reported, limiting the precision of derived analyses, and enrollment year was unavailable for approximately one-quarter of studies. Finally, as we did not assess study quality, sample size, or risk of bias, our analysis describes where evidence was generated, not its relative strength. Despite these limitations, the central finding is well supported. Across three major guidelines, most classifiable primary evidence supporting antiemetic therapy originated from high-income countries, with two countries alone contributing half of all studies. This pattern was consistent across all emetogenicity categories and is unlikely to be explained by misclassification of multinational trials as, even after excluding all 40 multinational trials, HICs still accounted for 78.1% (118/151) of trials. Furthermore, even under the most conservative assumption of classifying all 40 trials as LMICs, HICs would still remain the majority at 61.8% (118/191). The evidence base underlying global standards for CINV prophylaxis appears to be concentrated in high-income settings.

These findings support ongoing efforts to adapt antiemetic recommendations to resource-limited settings and highlight the importance of explicitly considering access, affordability, and local applicability when translating evidence into global recommendations [11].

## Conclusion

In a descriptive synthesis of the primary studies cited across the contemporary MASCC/ESMO [8], NCCN [9], and ASCO [10] antiemetic guidelines, we found that the evidence for CINV antiemetic prophylaxis is mostly derived from HICs and that LMICs are notably underrepresented at every level of chemotherapy emetogenicity. In many cases, this matters because the populations least represented in the evidence are the same populations with the least access to the antiemetics that evidence supports where current recommendations, even though they are supported in the settings where the trials were conducted, may not be completely generalizable to resource-limited settings. Addressing this gap will require generating antiemetic evidence in the settings where it is most needed, through noninferiority trials of regimens that are actually accessible in those regions. Until more context-specific data become available, clinicians in LMICs should apply CINV guidelines with the understanding that although the supporting evidence is robust, it has been generated predominantly in HIC settings.

## Supplementary information

Below is the link to the electronic supplementary material.

ESM 1 (DOCX 502 KB)

## Data Availability

No datasets were generated or analysed during the current study.
