# Supplementary material for: Global inequitable distribution of seminal studies of chemotherapy-induced nausea and vomiting
Source: Support Care Cancer. 2026 Aug 15;34(9):857. doi: 10.1007/s00520-026-11103-0 (PMC13476326; doi:10.1007/s00520-026-11103-0)
Supplement: Supplementary file 1 [file 520_2026_11103_MOESM1_ESM.docx]

**Supplemental Table 1.** Characteristics of all studies included (n=195)

| **Study (First author and year)** | **Country of Origin** | **LMIC vs HIC** | **Age - Median (IQR)** | **Age - Mean +/- SD** | **Percentage Female** | **Primary Cancer Type** | **Chemotherapy Regimen** | **Chemotherapy Emetogenicity** | **Antiemetic Regimen (5HT3, NK1, DEX, OLN, other)** |
| --- | --- | --- | --- | --- | --- | --- | --- | --- | --- |
| Aapro 1981 (19) | United States | HIC | 55 (Range: 21-83) | NR | 44.1% | NR | 1) cis-platinum-mitomycin-C-vincristine-bleomycin (2) cis-platinum-adriamy- cincyclophosphamide (3)cin-vinblastine (4) cis-platinum-adriamycin-cyclo- phosphamide-bleomycin-vincristine-mitomycin C (5) cis-platinum-vinblastine-bleomycin-hexamethyl- melamine (6) cis-platinum-5-fluorouracil-vin- blastine-bleomycin | HEC | DEX, Others (neuroleptics (droperidol and haloperidol), diphenhydramine, biperiden) |
| Aapro 1984 (21) | United States | HIC | Range 2 arms: 38-79, 22-76 | 56.5 | 58.8% | NR | cis-platinum, actinomycin D, imidazole carboxamide, high-dose CHOP (cyclo- phosphamide, doxorubicin, vincristine, prednisone), or MOP- BAP (nitrogen mustard, vincristine, prednisone-bleomycin,doxorubicin, prednisone) | HEC | DEX, other (metoclopramide) |
| Aapro 2006 (20) | Multinational (North America and Europe) | HIC | NR | 51.6 +/- 14.0 | 51.1% | Mixed (Ovarian, Lung, Gastric, Breast and Hodgkin's disease) | HEC (cisplatin, cyclophosphamide, carmustine, dacarbazine, or mechlorethamine) | HEC | 5HT3, DEX |
| Aapro 2010 (16) | Multinational (Austria, Germany, Italy and Spain) | HIC | NR | 51.6+/-10.11 | 100.0% | Breast cancer | MEC (cyclophosphamide and/or anthracyclines) | MEC | 5HT3, DEX |
| Aapro 2012 (17) | Multinational (Austria, Belgium, France, Italy, Spain, Sweden, The Netherlands, and UK) | HIC | NR | 56.7 +/-11.4 | 72.9% | Mixed (Breast, colorectal, lung, ovarian, other) | any one of the following: cisplatin, cyclophosphamide, dacarbazine, oxaliplatin, carboplatin, doxorubicin, epirubicin, irinotecan, oral temozolomide, oral vinorelbine | HEC | 5HT3, NK1, DEX, other (benzodiazepine, benzamides) |
| Aapro 2014 (18) + Jordan 2016 (107) | Multinational (Argentina, Belarus, Brazil, Bulgaria, Croatia, Germany, Hungary, India, Italy, Mexico, Poland, Romania, Russia, Ukraine and the United States) | HIC | 54 | NR | 98.0% | Breast cancer, other | HEC (cyclophosphamide, combine with doxorubicin or epirubicin) | HEC | NK1, 5HT3, DEX, other (metoclopramide rescue) |
| Abdel-Malek 2017 (22) | Egypt | LMIC | 45 (Range: 38-56) | NR | 46.7% | Diffuse B cell lymphoma (Non-Hodgkin's lymphoma) | Etoposide, cisplatin, solumedrol, cytarabine | HEC | 5HT3, NK1, DEX |
| Abe 2016 (23) | Japan | HIC | 57 (Range: 25-76) | NR | 100.0% | Gynecological Cancer | Cisplatin (paclitaxel or adriamycin) | HEC | OLN, 5HT3, NK1, DEX, other (metoclopramide used as rescue medication) |
| Adra 2016 (24) | United States | HIC | 33 (Range: 15-66) | NR | 0.0% | Germ cell tumours | Cisplatin combined with etoposide and bleomycin (BEP) | HEC | NK1, 5HT3, DEX, other (lorazepam, prochlorperazine, dexamethasone or promethazine additionally used as rescue medications) |
| Albany 2012 (25) | United States | HIC | 33 (Range: 16-62) | NR | 0.0% | Germ cell cancer | Cisplatin combined with etoposide and bleomycin (BEP) | HEC | 5HT3, NK1, DEX, other (lorazepam, prochlorperazine, or metoclopramide used as rescue meds) |
| Aridome 2016 (26) | Japan | HIC | NR | 65.0 +/- 10.0 | 43.4% | Colorectal cancer | Oxaliplatin‑ or irinotecan‑based MEC (FOLFOX, XELOX or FOLFIRI) | MEC | 5HT3, NK1, DEX |
| Arpornwirat 2009 (27) | Multinational | HIC | Ranges (6 arms): 22-83, 33-88, 20-85, 26-82, 22-84, 28-88 | 58.0 | 60.0% | Breast, non-small cell lung, colorectal, and ovarian cancers, other | Carboplatin, 5-Fluorouracil, Paclitaxel, Oxaliplatin, Doxorubicin, Cyclophosphamide, Gemcitabine, Docetaxel, Etoposide, Epirubicin | MEC | NK1, 5HT3, DEX |
| Audhuy 1996 (28) | NR | NR | NR | 54.6 +/-12.0 | 33.5% | Head and neck, lung, digestive system, and female reproductive organs, other | High-dose cisplatin | HEC | 5HT3 |
| Badalamenti 2019 (29) | Italy | HIC | 53 (Range: 25-80) | NR | 50.0% | Sarcoma/soft tissue | Epirubicin and Ifosfamide | MEC | NK1,5HT3 combination, DEX, other (metoclopramide as rescue medication) |
| Bloechl-Daum 2006 (30) | Multinational (Denmark, France, Italy, Germany, the United Kingdom, and the United States) | HIC | NR | 55.5 +/- 12.1 | 71.5% | Breast, lung cancer, other | HEC: Cisplatin, dacarbazine MEC: cyclophosphamide, doxorubicin, and/or epirubicin, carboplatin and other | HEC | 5HT3, DEX, other (unspecified) |
| Boccia 2011 (31) | Multinational (Europe, India, Mexico, and the USA) | HIC | NR | 54.5 +/- 13.5 | 51.5% | NR | Multi-day MEC or HEC regimens (cisplatin and non-cisplatin) | HEC | 5HT3, DEX |
| Bonneterre 1995 (32) | France | HIC | Granisetron then ondansetron: 50 (range 20-82); Ondansetron then granisetron: 53 (range 23-76) | NR | 90.0% | Breast cancer | ABV: adriamycin, bleomycin, vinblastine; BVD: adriamycin, bleomycin, vinblastine, dacarbazine; ACVBP: adriamycin, cyclophosphamide, vindesine, bleomycin, prednisone; ACVBPM: adriamycin, cyclophosphamide, vindesine, bleomycin, prednisone, methotrexate; CARBOVIN: carboplatin, vinorelbine; CC: carboplatin, cyclophosphamide; CHOP: cyclophosphamide, adriamycin, vincristine, prednisone; CHOPB1: cyclophosphamide, adriamycin, vincristine, prednisone, bleomycin; CMF: cyclophosphamide, methotrexate, fluorouracil; COP: cyclophosphamide, vincristine, prednisone; CVP: cyclophosphamide, teniposide, prednisone; EBVD: epirubicin, bleomycin, vinblastide, dacarbazine; ECVBP: epirubicin, cyclophosphamide, vindesine, bleomycin, prednisone; FEC: fluorouracil, epirubicin, cyclophosphamide; FEM: fluorouracil, epirubicin, mitomycin C; FNC: fluorouracil, novantrone, cyclophosphamide; FTC: fluorouracil, THP-adriamycin, cyclophosphamide; MBACOD: methotrexate, bleomycin, adriamycin, cyclophosphamide, vincristine, dexamethasone; MFN: methotrexate, fluorouracil, novantrone; MOPP: mechlorethamide, vincristine, procarbazine, prednisone; VINNOV: vinorelbine, mitoxantrone; VMN: vindesine, mitomycin C, novantrone. | HEC | 5HT3, other non-specified standard anti-emetic drugs for those failing to achieve control of nausea and vomiting |
| Bright 2024 (33) | India | LMIC | NR | 45.9 +/- 12 | 65.0% | Breast cancer, head and neck cancer, bladder cancer, osteosarcoma, testicular cancer, soft tissue sarcoma, male breast cancer, gallbladder cancer, nasopharyngeal cancer, uterine/endometrial cancer, and rhabdomyosarcoma | Chemotherapy regimens consisting of epirubicin, cisplatin, doxorubicin and cyclophosphamide, doxorubicin, dacarbazine, cyclophosphamide | HEC | DEX, 5HT3, OLN, other (capsaicin) |
| Bubalo 2018 (34) | United States | HIC | 2 arms Range: 19-63, 19-60 | 46.0 | 30.0% | Acute lymphoblastic leukemia, acute myelogenous leukemia, chronic lymphocytic leukemia, chronic myelogenous leukemia, non-Hodgkin lymphoma, myelodysplastic syndrome, myelofibrosis, and Waldenstrom’s macroglobulinemia | Myeloablative CY/total body irradiation (TBI) or targeted busulfan (TBu)/CY as their conditioning regimen | Minimal | NK1, 5HT3, DEX |
| Bubalo 2024 (35) | United States | HIC | Range: 23-77 | 55.0 | 37.0% | Diffuse large B-cell lymphoma, Hodgkin lymphoma, mantle cell lymphoma, and peripheral T-cell lymphoma | BEAM conditioning therapy (carmustine, etoposide, cytarabine, melphalan) | HEC | NK1/5HT3 combination, DEX, other unspecified rescue agents |
| Cao 2020 (36) | China | LMIC | 48 | NR | 100.0% | Breast cancer | Epirubicin plus cyclophosphamide, or cisplatin-containing regimens | HEC | NK1, 5HT3, DEX, other (mirtazapine) |
| Caracuel 2015 (37) | Spain | HIC | NR | 58+/-10.9 | 61.0% | Breast, Colorectal, Gynecologic, Lung, ENT, Pancreas, Gastric, and Others | Very low, low, moderate, high and very high emetogenicity risk regimes (cisplatin >=70mg/m2) | HEC | NK1, 5HT3, DEX, other (methylpredinisolone and metoclopramide) |
| Cass 2019 (38) | United States | HIC | 30 | 35.7+/-18.7 | 83.3% | Highlighted Cervical cancer, Ewing’s sarcoma, rhabdomyosarcoma, Hodgkin lymphoma, mesothelioma | Cisplatin, paclitaxel, bevacizumab, or vincristine, cyclophosphamide, doxorubicin or vincristine, irinotecan, or brentuximab vedotin, doxorubicin, vinblastine, dacarbazine, or cisplatin, pemetrexed | HEC | NK1, 5HT3, DEX, OLN, other (diphenhydramine, famotidine) |
| Cassileth 1983 (39) | United States | HIC | DEX: 52 (Range: 30-70), Placebo: 54 (29-66) | NR | 100.0% | Breast cancer | Cyclophosphamide, methotrexate sodium, fluorouracil, or Doxorubicin hydrochloride, vincristine sulfate, or Mitomycin | MEC | DEX, other (prochlorperazine) |
| Celio 2011 (44) + Celio 2012 (43) | Italy | HIC | Only one arm reported: (57.5) | 57.1 +/- 11.6 | 65.1% | Breast, colorectal, lung, other | AC-based, Oxaliplatin-based, Carboplatin-based, Irinotecan-based, Other | MEC | 5HT3, DEX, and other (metoclopramide as rescue) |
| Celio 2019 (45) | Italy | HIC | Across 3 groups: 57 (37-75), 62 (30-80), 64 (44–77) | NR | 100.0% | Cervical, endometrial, ovarian, other | Carboplatin plus paclitaxel | MEC | 5HT3, DEX, OLN, other (histamine receptor type 1 and type 2 blocking agents) |
| Celio 2021 (41) + Celio 2022 (42) + Celio 2022 (40) | Italy | HIC | Across 3 groups: 66 (44-79), 63 (34-77), 64 (40-76) | 63.3 +/-7.8 | 32.9% | Non-small cell lung cancer | Cisplatin-based (with Pemetrexed, Gemcitabine, Vinorelbine, Other) | HEC | NK1, 5HT3, DEX, and other (metoclopramide) |
| Chang 2020 (46) | Multinational (China, Taiwan, Thailand, Korea) | HIC | NR | 54.6 | 30.7% | Solid tumours, mostly lung cancer | Cisplatin, Gemcitabine/Gemcitabine HCl, Pemetrexed/Pemetrexed disodium, Docetaxel, Etoposide | HEC | 5HT3, NK1, DEX |
| Chawla 2003 (47) | Multinational (United States and other) | HIC | NR | 56 +/- 13.2 | 43.6% | Respiratory, urogenital, other | Cisplatin, Docetaxel, Paclitaxel | HEC | 5HT3, NK1, DEX |
| Cheng 2022 (48) | China | LMIC | 56 (25-81) | NR | 39.7% | Colorectal cancer | MEC - mFOLFOX6 (oxaliplatin, leucovorin, 5-fluorouracil) | MEC | 5HT3, NK1, DEX |
| Chevallier 1990 (49) | Multinational (France, Switzerland, U.K., West Germany) | HIC | NR | 55.5 | 37.2% | NR | Cisplatin, other cytostatic drugs allowed | HEC | 5HT3, DEX, other (metoclopramide) |
| Chevallier 1993 (50) | Multinational (France, Switzerland, UK, West Germany) | HIC | NR | 55.7 +/- 12.2 | 34.9% | NR | Cisplatin | HEC | 5HT3, DEX, other (metoclopramide) |
| Clemmons 2018 (51) | United States | HIC | 2 arms (ranges): (22-72), (22-74) | 55.0 | 40.6% | Multiple myeloma amyloid, Lymphoma, Acute myeloid leukemia, Myelodysplastic syndrome, Chronic myeloid leukemia, Chronic lymphocytic leukemia, Acute lymphoblastic leukemia, Other | ICE (ifosfamide, carboplatin, etoposide), 7 + 3 (cytarabine, anthracycline), ABVD (doxorubicin, bleomycin, vinblastine, dacarbazine), BEAM (carmustine, etoposide, cytarabine, melphalan), high-dose melphalan, BuCy (busulfan, cyclophosphamide), FluBu (fludarabine, busulfan), FluCy (fludarabine, cyclophosphamide) ± TBI, BuMel (busulfan, melphalan), and FluBuCy | HEC | 5HT3, NK1, DEX, OLN |
| Clemons 2020(52) | Canada | HIC | 2 arms (ranges): 23-88, 23-74 | 51.0 | 100.0% | Breast cancer | Anthracycline-cyclophosphamide or platinum-based chemotherapy (AC, FEC, TCH) | MEC | 5HT3, NK1, DEX, OLN |
| Crichton 2024(53) | Australia | HIC | NR | 59 +/- 8 | 68.0% | Breast, Lymphoma, Digestive, Lung, Urogenital, Gynecological, Other | MEC to HEC | HEC | 5HT3, NK1, DEX, other ginger root power, D2RA which is a dopamine receptor antagonist) |
| Cupissol 1990 (54) | France | HIC | NR | 57.6 | 28.6% | NR | Cisplatin | HEC | 5HT3, other (metoclopramide) |
| Dean 2020 (57) | United States | HIC | NR | 32+/-12.5 | 50.0% | Not a cancer population | NR | Minimal | OLN, other (metoclopramide, capsaicin cream) |
| DeMulder 1990 (55) | Netherlands | HIC | Two arms (range): 58 (33-74), 57 (27-74) | NR | 56.2% | Head or neck, Lung, Gastrointestinal, Genito-urinary, Gynecologic, Other | Cisplatin, Cisplatin and cyclophosphamide, Cisplatin and methotrexate with or without vinblastine, Cisplatin and podophyllotoxin derivatives, Cisplatin/5 and fluorouracil, Cisplatin and vindesine with or without bleomycin, Cisplatin, epidoxorubicin, and cyclophosphamide, Cisplatin, epirubicin, cyclophosphamide, and prednisolone, Other | HEC | 5HT3, other (metoclopramide) |
| deWit 2004 (56) + Poli-Bigelli 2003 (163) + Hesketh 2003 (90) + Hesketh 2006 (91) | Multinational (United States and unspecified outside of United States) | HIC | NR | 55.5 +/- 13.0 | 44.0% | Non-small cell lung cancer, other lung/respiratory, urogenital, other | Cisplatin (with or without docetaxel or paclitaxel) | HEC | 5HT3, DEX, NK1 |
| DiRenzo 2020(60) | Italy | HIC | 56.5 (48-62); Range: 24-78 | 54.0 +/- 10.8 | 37.8% | Non-Hodgkin’s lymphoma | DHAOX, DHAP, GIFOX, IEV, IGEV, IVAC, R-DHAOX, R-DHAP, R-EPOCH, R-ICE, R-IEV, R-MTX ARAC HD, BEAM (carmustine, etoposide, cytarabine, melphalan), FEAM (fotemustine, etoposide, cytarabine, melphalan), Melphalan/Mitoxatrone | HEC | NK1, 5HT3, other (metoclopramide) |
| DiRenzo 2020(58) | Italy | HIC | Two arms with ranges: 56 (19-78), 60.5 (20-81) | NR | 46.2% | Acute myeloid leukemia (AML), high-risk myelodysplastic syndrome | Daunorubicin + low-dose cytarabine, Cytarabine + fludarabine + liposomal doxorubicin, Cytarabine + fludarabine + idarubicin, Cytarabine + fludarabine | MEC | 5HT3, NK1, other (rescue medications allowed as needed) |
| DiRenzo 2022(59) | Italy | HIC | 56.5 (48-62); Range: 24-78 | 54.0 +/- 10.8 | 37.8% | Relapsed/refractory aggressive NHL (Diffuse Large B-Cell Lymphoma, Mantle Cell Lymphoma, Primary Mediastinal B-Cell Lymphoma, Peripheral T-Cell Lymphoma | FEAM, BEAM,Melphalan/Mitoxantrone | Minimal | NK1, 5HT3, other (metoclopramide) |
| Ebrahimi 2020 (61) | Iran | LMIC | NR | 50.0 +/- 12.8 | 69.8% | Breast cancer, Colorectal cancer, Lymphoma, Ovarian cancer, Lung cancer, Prostate cancer, Gastric cancer, Other | Epirubicin or doxorubicin and cyclophosphamide, paclitaxel and carboplatin, docetaxel | MEC | NK1, 5HT3, DEX, other (metoclopramide) |
| Einhorn 2007(62) | United States | HIC | NR | 33.3 +/- 10.0 | 0.0% | Testicular cancer (germ cell tumours) | BEP (Bleomycin/etoposide/cisplatin), EP (etoposide/cisplatin), VIP (Etoposide/ifosfamide/cisplatin) | HEC | 5HT3, DEX |
| Eisenberg 2003 (63) | Multinational (United States and Mexico) | HIC | NR | 54.0 +/- 13.0 | 82.1% | Breast carcinoma, lung carcinoma, non-Hodgkin lymphoma | Anthracycline + cyclophosphamide, Carboplatin-based, Cyclophosphamide-based, Cisplatin, Other | HEC | 5HT3, DEX, other (methylprednisolone) |
| Eisenberg 2004 (64) | United States | HIC | NR | 60.0 +/- 11.0 | 20.0% | NR | HEC: Cisplatin, cyclophosphamide | HEC | 5HT3, other (rescue medication allowed) |
| Fauser 1996 (65) | Multinational (Europe) | HIC | NR | 53.2 +/- 13.0 | 61.2% | Breast cancer, lung cancer, other | Moderately emetogenic drugs (carboplatin, cyclophosphamide, doxorubicin, epirubicin, dacarbazine, mustine, or ifosfamide) | HEC | 5HT3 |
| Fauser 2000 (66) | Multinational (Europe) | HIC | NR | 45.0 | 20.5% | Testicular cancer, Lung, Head/neck, Gynaecological tumours, Gastrointestinal, Breast cancer, Lymphoma, Other | Cisplatin, etoposide, 5-FU, bleomycin, or ifosfamide | HEC | 5HT3, DEX, other (rescue medication allowed) |
| Fox 1993 (67) | United States | HIC | 2 Arms (ranges): 28 (19-42), 25 (15-24) | NR | 2.3% | Germ cell tumours, small-cell lung cancer | Cisplatin, etoposide, bleomycin, ifosfamide | HEC | 5HT3, DEX, other (chlorpromazine) |
| Furukawa 2015 (68) | Japan | HIC | 2 arms (ranges): 62 (43-83), 59 (36-76) | NR | 100.0% | Ovarian, cervical, endometrial cancer | Paclitaxel and carboplatin | MEC | 5HT3, DEX, other (Diphenhydramine, Ranitidine, and Metoclopramide for rescue) |
| Gao 2013 (70) | China | LMIC | 52.8 (Range: 21-74) | NR | 41.5% | Lung, Nasopharyngeal carcinoma, Testicular cancer, Esophagus, Stomach cancer, Oropharyngeal cancer | Cisplatin based | HEC | NK1, 5HT3, DEX, other (rescue medication allowed) |
| Gao 2022 (71) | China | LMIC | NR | 59.2 +/- 8.5 | 47.5% | Lung cancer, others | Cisplatin combined with others (gemcitabine, docetaxel, etoposide, pemetrexed, paclitaxel, capecitabine, irinotecan, bevacizumab, Herceptin) | HEC | OLN, NK1, 5HT3, DEX |
| Gao 2023 (69) | China | LMIC | 2 arms: 64, 65 | NR | 37.8% | Breast, Lung/respiratory, Ovarian, Colorectal, Gastric, Esophageal, Pancreatic, Other | Carboplatin, cisplatin, epirubicin | HEC | NK1, 5HT3, DEX |
| Gebbia 1994 (72) | Italy | HIC | NR | 57.4 | 57.1% | Head and neck, Lung, Urinary bladder, Ovary, Stomach, Endometrium, Vulva, Breast, Testis, Sarcoma, Breast, Lung, Ovary, Stomach, Non-Hodgkin lymphoma, Melanoma | Cisplatin, cyclophosphamide, epidoxorubicin, vinca alkaloids, doxorubicin, methotrexate, 5-fluorouracil, ifosfamide, vinorelbine | HEC | 5HT3 |
| Giralt 2011 (73) | United States | HIC | NR | 58.5 | 35.6% | Multiple myeloma | Melphalan | Minimal | 5HT3, DEX |
| Gonullu 2012 (74) | Turkey | LMIC | NR | 53.6+/-1.63 | 36.0% | Solid-organ tumours | MEC to HEC | HEC | 5HT3 |
| Gralla 1981 (77) | United States | HIC | 4 arms: 53 (39-73), 55 (21-66), 60 (47-66), 62 (35-69) | NR | 19.5% | Bronchogenic carcinoma, esophageal carcinoma, head-and-neck carcinoma, synovial sarcoma | Cisplatin, vindesine | HEC | Other (Metoclopramide, prochlorperazine) |
| Gralla 2003 (75) | Multinational (Germany, Italy, UK, The Netherlands, and Russia) | HIC | NR | 55.4 | 72.1% | Breast, lung, bladder, colon, rectal, small-cell lung, and gastric cancer | Cyclophosphamide, doxorubicin, cisplatin, methotrexate, carboplatin, epirubicin, idarubicin, ifosfamide, irinotecan, mitoxantrone | HEC | 5HT3 |
| Gralla 2005 (76) | NR | NR | NR | NR | 42.0% | Solid-organ tumours | Cisplatin, doxorubicin, cyclophosphamide, docetaxel, paclitaxel, etoposide, fluorouracil, gemcitabine, vinorelbine | HEC | 5HT3, NK1, DEX |
| Grote 2006 (78) | United States | HIC | 59.5 (Range: 35-84) | NR | 78.0% | Breast, colorectal, lymphoma, lung, other | Cyclophosphamide, anthracycline, carboplatin, oxaliplatin, epirubicin, idarubicin, ifosfamide, irinotecan, mitoxantrone, methotrexate, doxorubicin, cisplatin | HEC | 5HT3, NK1, DEX |
| Grunberg 2004 (80) | Multinational (Denmark, France, Italy, Germany, U.K, United States) | HIC | 56.0 (Range: 19.0-87.0) | 55.5+/-12.1 | 71.5% | Breast carcinoma, lung carcinoma, other | Cisplatin, DTIC (dacarbazine), cyclophosphamide, doxorubicin, epirubicin, carboplatin, other | HEC | 5HT3, DEX |
| Grunberg 2009 (81) | United States | HIC | 51 (Range: 33-74) | NR | 97.6% | Breast cancer | Cyclophosphamide, doxorubicin, epirubicin, docetaxel, 5-fluorouracil, trastuzumab, triptorelin | MEC | 5HT3, NK1, DEX |
| Grunberg 2011 (79) | Multinational | HIC | 2 arms: 56 and 57 | 55.6 | 36.7% | Lung cancer, GI cancer, reproductive or genitourinary cancer, renal and urinary tract cancer, breast cancer | Cisplatin | HEC | 5HT3, NK1, DEX |
| Hafermann 2011 (82) | United States | HIC | NR | 54.4 +/- 15.7 | 50.0% | Not a cancer population | NR | Minimal | 5HT3 |
| Hamada 2014 (83) | Japan | HIC | NR | 33.9+/- 8.3 | 0.0% | Testicular germ cell tumour | BEP (Bleomycin, etoposide, cisplatin), EP (Etoposide, cisplatin), VIP (Etoposide, ifosfamide, cisplatin) | HEC | 5HT3, NK1, DEX |
| Hashimoto 2020 (84) | Japan | HIC | Two arms: 65(22-75) and 66(30-75) | NR | 33.0% | Head and neck, lung, esophageal, gastric, gynaecological, urological, other | Cisplatin | HEC | OLN, NK1, 5HT3, DEX |
| Hata 2022 (85) | Japan | HIC | Two arms: 67 (40-81) and 66 (33-82) | NR | 23.2% | Lung, Esophagus, Head and neck, Other | Cisplatin HEC | HEC | 5HT3, NK1, DEX |
| Herman 1979 (86) | United States | HIC | 33 (range: 15-74) | NR | NR | Testicular carcinoma, non-Hodgkin's lymphomas, Hodgkin's disease | Cisplatin, vinblastine, bleomycin, CHOP, MOPP, actinomycin D, dacarbazine, 5-fluorouracil, melphalan, nitrosourea compounds | HEC | Other (Nabilone; Prochlorperazine) |
| Herrstedt 2007 (87) | Denmark | HIC | 2 arms: 34 (Range: 18-60) and 34 (18-62) | NR | 6.1% | Germ cell tumours | Cisplatin, etoposide, bleomycin | HEC | 5HT3, , Other (Metopimazine) |
| Herrstedt 2019 (88) | Multinational (UK, Germany, Denmark) | HIC | 5 arms (range): 57 (27-78), 58 (32-79), 59 (30-75), 57 (32-79), 58 (37-79 | NR | 78.4% | Breast cancer | Cisplatin, Anthracycline-cyclophosphamide, epirubicin, doxorubicin | HEC | 5HT3, NK1, DEX, other (amisulpride) |
| Hesketh 1996 (89) | United States | HIC | 62 | NR | 38.1% | Lung, Gastrointestinal, Gynecologic, Head/neck, Other | Cisplatin | HEC | 5HT3 |
| Hesketh 2018 (92) + Hesketh 2014 (93) | Multinational | HIC | NR | 55.5 +/- 9.5 | 42.9% | Lung and respiratory tract cancer, Bladder, Breast, Colorectal, Gastric, Other GI, Gynecologic, Other urogenital, Head and neck, Rectal, Other | Cisplatin, carboplatin | HEC | 5HT3, NK1, DEX |
| Hickok 2005 (95) | United States | HIC | NR | 52.7 | 94.3% | Breast cancer, Lymphoma, Urinary bladder cancer, multiple myeloma, endometrial cancer, and soft-tissue sarcoma | Doxorubicin containing regimens | MEC | 5HT3, DEX, other (prochlorperazine, methyprednisolone) |
| Iihara 2020 (96) | Japan | HIC | 58(Range:34-79) | NR | 100.0% | Ovarian cancer, Cervical cancer, Endometrial cancer, and Others | Carboplatin combined with paclitaxel or docetaxel | MEC | 5HT3, NK1, DEX, OLN |
| Inoue 2017 (98) | Japan | HIC | 58(Range:30-74) | NR | 28.8% | Lung cancer | Carboplatin combined regimens (PEM/pemetrexed, BEV/ bevacizumab, PTX/ paclitaxel, nabPTX/nab-paclitaxel, ETP/etoposide, S1/Tegafur/Gimeracil/Oteracil, GEM/gemcitabine) | MEC | 5HT3, NK1, DEX, other (metoclopramide, prochlorperazine, diphenhydramine salicylate, Tsukushi AM combination powder) |
| Ioroi 2018 (99) | Japan | HIC | NR | 38.9+/-10.6 | 0.0% | Testicular germ cell tumours (Seminoma and non-seminoma) | Cisplatin-based combination (BEP, VIP, TIP) | HEC | 5HT3, NK1, DEX |
| Isoda 2017 (100) | Japan | HIC | 56 (range: 44-65) | NR | 33.3% | Multiple myeloma | High-dose melphalan | Minimal | 5HT3, NK1, DEX, OLN, other (metoclopramide) |
| Italian Group for Antiemetic Research 2004 (15) | Multinational (Italy and Yugoslavia) | HIC | 3 arms: 56, 51, 52 | NR | 97.3% | Breast and other | Cyclophosphamide, doxorubicin, epirubicin, or carboplatin | MEC | 5HT3, DEX, other (metoclopramide, prochlorperazine) |
| Italian Research Group 1995 (14) | Italy | HIC | 61 | NR | 31.7% | Ovary, Lung, Head-neck, Bladder, and Other | Cisplatin containing chemotherapy (Anthracyclines ± others, Low emetogenic chemotherapy, 5-fluorouracil, Cyclophosphamide) | HEC | 5HT3, DEX, other (metoclopramide, diphenhydramine) |
| Ithimakin 2020 (101) | Thailand | LMIC | 3 arms (range): 52.5 (27.3, 73.7), 53.7 (24.2, 71.5), 55.7 (26.6, 79.4) | NR | 78.6% | Breast, head and neck, lung, and others | Doxorubicin,cyclophosphamide or Cisplatin | HEC | 5HT3, DEX, NK1, OLN |
| Ito 2018 (102) + Celio 2022 (40) | Japan | HIC | 2 arms (range): 54.1 (27-78), 55.0 (24-79) | NR | 80.8% | Breast, Esophageal, Gastric, Lung, and Others | Anthracycline plus cyclophosphamide or Cisplatin-containing regimens | HEC | 5HT3, NK1, DEX, OLN, other (prochlorperazine, metoclopramide, domperidone, alprazolam, lorazepam) |
| James 2017 (103) | India | LMIC | NR | NR | 91.4% | Breast cancer, Ovarian cancer, Lung cancer, Rectal cancer, Other cancers | Carboplatin, cyclophosphamide, adriamycin, cisplatin, paclitaxel, capecitabine, gemcitabine | HEC | 5HT3, NK1, other (lorazepam, domperidone) |
| Jantunen 1993 (104) | Finland | HIC | Range: 24-81 | 50.6 | 83.7% | Breast, Gastrointestinal, Lymphoma, Lung, Head and neck, Mesothelioma, Other | CMF, FAC/FEC, Cyclophosphamide+mitoxantrone+5-FU, Other cyclophosphamide-containing, Doxorubicin/epirubicin+MTX+5-FU, Other anthracycline-containing, Carboplatin-containing, Mitomycin+MTX+mitoxantrone, DTIC-containing, Cisplatin, Other | HEC | 5HT3, other (metoclopramide) |
| Jeon 2019 (105) | Korea | HIC | 2 arms: 60 and 63 | NR | 16.7% | Colorectal, stomach, lung, other | Oxaliplatin containing, Irinotecan containing, Carboplatin containing | MEC | 5HT3, DEX, OLN |
| Jeong 2016 (106) | Korea | HIC | 59 (Range: 36-81) | NR | 28.6% | Esophagus, stomach, colorectal, hepatobiliary | HEC or MEC | HEC | 5HT3, NK1, DEX |
| Jordan 2009 (108) | Germany | HIC | 2 arms (ranges): (22-71), (18-67) | 40.4 | 19.2% | Sarcoma, Germ cell cancer, Myeloma, Lymphoma, Primary unknown (CUP), Thymic cancer | HEC or MEC emetic risk (cisplatin/ifosfamide containing) | HEC | 5HT3, NK1, DEX |
| Kang 2020 (109) | Korea | HIC | NR | 59.9 +/- 11.9 | 38.0% | Lung and thymus, Breast, Head and neck, GU and GY, Gastrointestinal, Other | HEC (cisplatin-based, non-cisplatin) | HEC | 5HT3, NK1, DEX |
| Karthaus 2015 (110) | Multinational (United States, Latin America, Europe, Asia, and commonwealth of independent states) | HIC | NR | 57.9 +/- 9.7 | 41.0% | Gastric, Head and neck, Lung/respiratory tract, Ovarian, Bladder, Other | Cisplatin-based highly emetogenic chemotherapy | HEC | 5HT3, DEX |
| Karthaus 2020 (111) | Multinational (Belarus, Bosnia and Herzegovina, Bulgaria, Georgia, Greece, Hungary, Lithuania, Romania, Russian Federation) | HIC | NR | 59.4 +/- 8.6 | 33.0% | Lung/respiratory tract, Head and neck, Ovarian, Gastric, Bladder, Other | HEC (cisplatin, cyclophosphamide, carmustine, dacarbazine, or mechlorethamine). Concomitant chemotherapy days had etoposide, gemcitabine and fluorouracil as well. | HEC | 5HT3, DEX |
| Kaushal 2015 (112) | India | LMIC | 2 arms (range): 52 (36-70), 51 (34-69) | NR | 13.3% | Oral cavity, Oropharynx, Nasopharynx, Larynx, Hypopharynx | Docetaxel, carboplatin, and 5-Fluorouracil | MEC | 5HT3, NK1, DEX |
| Kim 2015 (113) | Korea | HIC | NR | 59.0 +/- 11.0 | 31.8% | Lung, Lymphoma, Stomach, Head and Neck, Breast, Esophagus, Hepatobiliary and pancreas, Bladder cancer, chondrosarcoma, leiomyosarcoma, skin melanoma, malignant neoplasm of retroperitoneum, skin cancer, neuroendocrine carcinoma, pleural mesothelioma, and thymic carcinoma | Cisplatin-containing highly emetogenic chemotherapy or non-cisplatin based | HEC | 5HT3, NK1, DEX |
| Kim 2017 (114) | Korea | HIC | NR | 60.3 +/- 11.4 | 45.2% | Gastrointestinal, Lung, Gynecologic, Other | Moderately emetogenic chemotherapy (MEC), non-anthracycline plus cyclophosphamide (AC)-based regimens including Carboplatin-based, Oxaliplatin-based, Irinotecan-based | MEC | NK1, 5HT3, DEX |
| Kimura 2015 (115) | Japan | HIC | 15-70 | 43.4 | 50.0% | Osteosarcoma, malignant fibrous histiocytoma, synovial sarcoma, leiomyosarcoma, rhabdomyosarcoma, dedifferentiated liposarcoma, myxoid liposarcoma, clear cell sarcoma | AP regimen (cisplatin and doxorubicin), IE regimen (ifosfamide and etoposide), AI regimen (ifosfamide and doxorubicin) | HEC | NK1, DEX, 5HT3 |
| Kitayama 2015 (116) | Japan | HIC | NR | 60.0 +/- 14.0 | 63.0% | Colorectal, Breast, Other | Moderately emetogenic chemotherapy (MEC): Oxaliplatin-based (mFOLFOX6, XELOX), Irinotecan-based (FOLFIRI), Carboplatin-based, docetaxel plus cyclophosphamide | MEC | 5HT3, DEX, NK1 |
| Komatsu 2015 (117) | Japan | HIC | NR | 64.0 | 43.3% | NR | Non-AC moderately-emetogenic chemotherapy: Oxaliplatin-based, Irinotecan-based, Carboplatin-based | MEC | 5HT3, DEX, other (antidopaminergic agents, phenothiazine tranquilizers, antihistamines, and benzodiazepines allowed as rescue drugs) |
| Kosaka 2016 (118) | Japan | HIC | NR | 53.1 +/- 10.7 | 100.0% | Breast cancer | Anthracycline-cyclophosphamide combination regimen. AC (doxorubicin and cyclophosphamide), EC (epirubicin and cyclophosphamide), FEC (5FU, epirubicin, and cyclophosphamide) | MEC | 5HT3, NK1, DEX |
| Kris 1985 (119) | United States | HIC | NR | 54.2 | 33.8% | Lung, other | Cisplatin, vindesine, vinblastine, mitomycin | HEC | DEX, other (metoclopramide, diphenhydramine, lorazepam) |
| Kris 1987 (120) | United States | HIC | 2 arms: 54 (35-73), 56 (31-69) | NR | 34.1% | Non-small cell lung, other | Cisplatin, vindesine, vinblastine, mitomycin | HEC | DEX, other (lorazepam, diphenhydramine, metoclopramide) |
| Kusagaya 2015 (121) | Japan | HIC | 2 arms: 70 (57-90), 73 (43-84) | NR | 28.8% | Non-small-cell lung cancer (Adenocarcinoma, Squamous cell carcinoma, Other) | Carboplatin-based (Carboplatin + paclitaxel +/- bevacizumab, Carboplatin + pemetrexed +/- bevacizumab, Carboplatin + S-1) | MEC | 5HT3, DEX, NK1 |
| Li 2019 (122) | China | LMIC | NR | 49.6 +/- 7.7 | 100.0% | Breast cancer | Anthracycline (pirarubicin or epirubicin) and cyclophosphamide | MEC | NK1, 5HT3, DEX |
| Liu 2015 (124) | China | LMIC | NR | 52.1 +/- 10.8 | 40.2% | Lung cancer, breast cancer, colorectal cancer, lymphoma, ovarian cancer, stomach cancer, oesophageal cancer, teratoma, oropharyngeal cancer, thymus cancer, cervical cancer, gingival cancer, malignant melanoma, laryngeal cancer, and glioblastoma | Moderately emetogenic (oxaliplatin, carboplatin, epirubicin, adriamycin) or highly emetogenic (cisplatin, dacarbazine) | HEC | OLN, 5HT3, DEX |
| Liu 2022 (123) | China | LMIC | NR | 59.6 +/- 9.6 | 39.5% | Lung cancer, others | Cisplatin-based combined with gemcitabine, docetaxel, etoposide, pemetrexed, paclitaxel, capecitabine, or irinotecan (sometimes with bevacizumab) | HEC | OLN, NK1, 5HT3, DEX |
| Lofters 1997 (125) | Multinational (Canada and United States) | HIC | NR | 55.0 +/- 12.9 | 70.5% | Breast, lung, gynecologic, other | Moderately emetogenic chemotherapy (Doxorubicin, Epirubicin, Cisplatin, Carboplatin, Cyclophosphamide). Examples given include AC, CAV, CMF, CAF, and etoposide + cisplatin | HEC | 5HT3, DEX |
| Longo 2011 (126) | Italy | HIC | 62 (Range: 22-82) | NR | 23.4% | Lung, Head and neck, Stomach, Bladder, oesophagus, mesothelioma, anus | Cisplatin-based highly emetogenic chemotherapy with concurrent docetaxel, gemcitabine, vinorelbine, etoposide, pemetrexed, 5-fluorouracil, or epirubicin | HEC | 5HT3, NK1, DEX, other (metoclopramide as rescue) |
| Maeda 2021 (127) | Japan | HIC | 71 (53-77) | NR | 42.2% | Lung cancer, endometrial cancer, ovarian cancer | Carboplatin-based chemotherapy plus Pemetrexed, Nab-paclitaxel, Paclitaxel, or Docetaxel (with or without Bevacizumab, Pembrolizumab, Atezolizumab) | MEC | OLN, NK1, 5HT3, DEX, other (metoclopramide and prochlorperazine) |
| Maehara 2015 (128) | Japan | HIC | NR | 58.4 +/- 12.3 | 100.0% | Ovarian cancer, Endometrial cancer | Paclitaxel and carboplatin | MEC | NK1, 5HT3, DEX |
| Malik 1995 (129) | Pakistan | LMIC | NR | 49.4 | 47.2% | Head and neck, Gynecologic, Esophageal, Testicular, Others | Cisplatin as a single agent or as part of combination therapy (CDDP + 5FU, CDDP + ifosfamide, CDDP + VP-16, CDDP + others) | HEC | DEX, other (metoclopramide, clemastine, lorazepam) |
| Mantovani 1996 (130) | Italy | HIC | NR | 58.2 +/- 9.9 | 3.4% | Head and neck cancer (Oral cavity, Oropharynx, Hypopharynx, Larynx, Maxillary sinus, Upper oesophagus). | Cisplatin, 5-fluorouracil, vinorelbine | HEC | 5HT3 |
| Martoni 1996 (131) | Italy | HIC | 62 (Range: 32-77) | NR | 25.0% | NSCLC, Bladder, Ovary, Others | Cisplatinum with one or two more drugs including epirubicin, cyclophosphamide, methotrexate, vinorelbine | HEC | 5HT3 |
| Marty 1990 (132) | Multinational (France, Germany, Switzerland, Belgium) | HIC | NR | 55.2 | 65.4% | NR | Carboplatin, Cisplatin, Cyclophosphamide, Dacarbazine, Doxorubicin, Epirubicin, Mustine (nitrogen mustard) | HEC | 5HT3, DEX, other (chlorpromazine) |
| Marty 1990 (133) | NR | Unknown | NR | NR | 57.7% | Gynecologic, Lung, Gastrointestinal, Genitourinary, Breast, Bone or soft tissue, Other | Cisplatin | HEC | 5HT3, other (metoclopramide) |
| Matsumoto 2020 (134) | Japan | HIC | 54; 2 arms ranges (27-82), (30-74) | NR | 100.0% | Breast | Anthracycline and cyclophosphamide regimen (AC-based regimen) such as doxorubicin + cyclophosphamide (AC), epirubicin + cyclo-phosphamide (EC), or 5-fluorouracil (5FU) + AC (FAC) or EC (FEC) | MEC | 5HT3, DEX, NK1 |
| Matsuura 2015 (136) | Japan | HIC | NR | 57.2 +/- 11.1 | 100.0% | Ovarian, Endometrial, Cervical | Carboplatin plus paclitaxel (TC regimen) | MEC | 5HT3, DEX |
| Matsuura 2022 (135) | Japan | HIC | 56; 2 arms ranges (34-79), (30-77) | NR | 100.0% | Breast | Doxorubicin-cyclophosphamide or Epirubicin-cyclophosphamide (AC/EC) | MEC | NK1, 5HT3, DEX |
| Minatogawa 2024 (137) + Shimomura 2021 (187) | Japan | HIC | 2 arms (ranges): 63 (35-74), 64 (25-74) | NR | 30.9% | Esophageal, Head and neck, Lung, Gastric, Others | Cisplatin-based | HEC | DEX, 5HT3, NK1, OLN |
| Miura 2013 (138) | Japan | HIC | 64 (range:36-78) | NR | 34.3% | Non-small cell lung cancer (Adenocarcinoma, Squamous cell carcinoma, Large-cell carcinoma, Not otherwise specified), Small cell lung cance | Cisplatin-based doublet chemotherapy (Cisplatin/Pemetrexed, Cisplatin/Vinorelbine, Cisplatin/Gemcitabine, Cisplatin/Irinotecan, Cisplatin/Etoposide, Cisplatin/Docetaxel) | HEC | 5HT3, DEX, NK1 |
| Molassiotis 2014 (139) | Multinational (France, Italy, Spain, Belgium, Sweden, U.K., Austria, and The Netherlands) | HIC | NR | 56.7 +/- 11.4 | 72.9% | Breast, Colorectal, Lung, Ovarian, Other | HEC, anthracyclines plus cyclophosphamide, MEC | HEC | NR |
| Morrow 1982 (140) | United States | HIC | 53 (range: 19-76) | NR | 70.0% | Breast, Hematologic, Lung, Other | NR | Minimal | Other (standard antiemetics such as prochlorperazine) |
| Mukhopadhyay 2017 (142) | India | LMIC | NR | 54.4 +/- 1.5 | 42.0% | Head and neck (HNCa), Cervix, Oesophagus, Pancreas, Ovary, Others | Platinum-based chemotherapy (Cisplatin, Carboplatin, Oxaliplatin) | HEC | 5HT3, DEX, OLN |
| Mukhopadhyay 2021 (141) | India | LMIC | NR | 52.0 +/- 11.5 | 62.7% | NR | HEC, MEC | HEC | OLN, 5HT3, DEX, NK1, other (metoclopramide) |
| Murphy 2023 (143) | United States | HIC | NR | 26.9 +/- 5.2 | 100.0% | Not a cancer population | NR | Minimal | 5HT3, other (metoclopramide, topical capsaicin cream) |
| Musso 2009 (144) | Italy | HIC | 2 arms (ranges): 51 (15-80), 50 (20-77) | NR | 38.5% | Non-Hodgkin lymphoma, Hodgkin disease, Acute myeloid leukemia, Solid tumor | DHAP, Hyper-CVAD, FluCy, HD-Mtx-AraC, BEACOPP, IGEV, ICE, FLANG, HD-AraC | Minimal | 5HT3, DEX, other (metoclopramide) |
| Nakagaki 2017(145) | Australia | HIC | 3 arms (ranges): 53 (26-65), 58 (20-68), 51 (21-65) | NR | 37.1% | Hematopoietic cancers | BEAM, FluMel, HDM, CyTBI, FluCy, Other | Minimal | 5HT3, NK1, OLN, Other (Metoclopramide and Lorazepam as rescue) |
| Nakamura 2017 (146) | Japan | HIC | 66 (range: 28-87) | NR | 37.8% | Gastric cancer, Colorectal cancer, Pancreas cancer, Biliary cancer | FL/FOLFOX/FOLFIRI +/- Bmab/Pmab, FOLFOX + Cmab, FOLFIRINOX, CapeOX/SOX +/- Bmab, Cape/CDDP + Tmab, Gem +/- E/nabPTX, Gem + CDDP, Weekly PTX, DTX + S-1 | Minimal | DEX, Other (Betamethasone and Hydrocortisone sodium succinate as rescue) |
| Nakashima 2017 (147) | Japan | HIC | 64 (range: 36-75) | NR | 23.3% | Non-small cell lung cancer, Small cell lung cancer, Malignant pleural mesothelioma, Thymoma | Cisplatin combined with Pemetrexed, Etoposide, Vinorelbine, Irinotecan, S-1, or Gemcitabine | HEC | 5HT3, NK1, DEX, OLN |
| Navari 1994 (152) | United States | HIC | NR | 59+/-11 | 30.4% | Lung, Head and neck, other | Cisplatin combined with Anthracyclines, Folic acid analogs and derivatives, Alkylators and analogs, Podophyllotoxin derivatives, Pyrimidine analogs, Vinca alkaloids and analogs, or Other | HEC | 5HT3, other (Conventional therapy/metoclopramide for rescue) |
| Navari 1995 (148) | United States | HIC | NR | 62.3 | 36.5% | Lung cancer, others | Cisplatin combined with etoposide, fluorouracil, vinblastine sulfate, or doxorubicin | HEC | 5HT3, Other (Metoclopramide, dexamethasone, and diphenhydramine as rescue medications) |
| Navari 2005 (150) | United States | HIC | 58.5 (Range: 25-84) | 55.7 +/- 14.3 | 76.7% | Bladder, Breast, Colon, Endometrium, Hodgkin’s lymphoma, Lung Non-small cell, Lung Small cell, Vaginal squamous cell | Cisplatin, Cisplatin/doxorubicin, Doxorubicin, Doxorubicin/cyclophosphamide, Irinotecan | HEC | 5HT3, DEX, OLN |
| Navari 2007 (149) | United States | HIC | 61 (36-84) | 60.9 +/- 10.3 | 55.0% | Bladder, Breast, Colon, Lung Nonsmall Cell, Lung Small Cell, Malignant lymphoma | Cisplatin, Carboplatin, Doxorubicin, Cyclophosphamide, Oxaliplatin | HEC | 5HT3, DEX, OLN |
| Navari 2011 (151) | United States | HIC | 2 arms (range): 63(39-77), 61(42-81) | NR | 68.0% | Bladder, Breast, Lung (non-small cell), Malignant lymphoma | Cisplatin, Doxorubicin and cyclophosphamide | HEC | 5HT3, NK1, DEX, OLN |
| Navari 2013 (153) | United States | HIC | 62 (Range: 38-79 years) | NR | 53.7% | Bladder, Breast, Lung (non-small cell), Malignant lymphoma | Cisplatin, doxorubicin, and cyclophosphamide | HEC | 5HT3, NK1, DEX, OLN, other (metoclopramide) |
| Navari 2016 (156) | United States | HIC | 3 arms (range): 58.0 (29.0-86.0), 56.0 (28.0-89.0), 57.0 (28.0-89.0) | NR | 72.4% | Breast, Lung, Other | Cisplatin-containing regimen, Anthracycline and cyclophosphamide | HEC | 5HT3, NK1, DEX, OLN |
| Navari 2016 (154) | United States | HIC | 2 arms (range): 63 (59-76), 61 (52-71) | NR | 22.8% | Head and neck, Esophageal | Cisplatin and 5-fluorouracil | HEC | 5HT3, NK1, DEX, OLN |
| Navari 2020 (155) | United States | HIC | Range: 39-79 | 63.0 | 53.3% | NR | NR | Minimal | OLN, other unspecified drugs |
| Nikbakht 2021 (157) | Iran | LMIC | Range: 16-87 | 52.65 +/- 13.98 | 56.0% | Breast, Colon, Stomach, Lymphoma, Ovarian, Lung, Other | FOLFOX, AC, Paclitaxel, CHOP_R, PC, Other | LEC | 5HT3, NK1, DEX, other (ranitidine, chlorphenramine, metoclopramide) |
| Noble 1994 (159) | Multinational (Austria, France, Germany, Netherlands, South Africa, UK) | HIC | 2 arms (range): 39-118, 38-114 | 51.8 | 22.6% | Head and neck, Lung, Ovary/cervix, Testis, Other | Cisplatin, Ifosfamide | HEC | 5HT3 |
| Olver 2013(160) | Australia | HIC | 30 (Range: 19-60 years) | NR | 4.0% | Germ cell tumors (testicular, retroperitoneal, mediastinal, or ovarian primary) | BEP, Accelerated BEP, EP, VIP | Minimal | 5HT3, NK1, DEX, other (lorazepam, metoclopramide, haloperidol, prochlorperazine) |
| Ostwal 2024 (161) | India | LMIC | 51 (Range: 19-80) | NR | 36.0% | Colorectal, Gastric or gastroesophageal, Non-small cell lung carcinoma, Biliary tract carcinoma, Urinary bladder cancer, Others | Capecitabine and oxaliplatin; leucovorin calcium, fluorouracil, and oxaliplatin; pemetrexed plus carboplatin; paclitaxel plus carboplatin; folinic acid, fluorouracil, and irinotecan hydrochloride; capecitabine and irinotecan hydrochloride; gemcitabine and oxaliplatin | MEC | 5HT3, NK1, DEX, OLN |
| Passik 2002 (162) | United States | HIC | NR | 58 +/- 16.8 | 73.3% | Breast, Lung, Ovary, Lymphoma, Myeloma, Pancreas, Bladder, Unknown | NR | Minimal | OLN |
| Radhakrishnan 2019 (164) | India | LMIC | 2 arms (range): 6(1-12), 5 (1-12) | NR | 39.0% | Acute lymphoblastic leukemia, Non-Hodgkin lymphoma, Hodgkin Lymphoma, Osteosarcoma, Ewing sarcoma, Wilms tumor, Neuroblastoma, Rhabdomyosarcoma, Others | ABVD, Cyclophosphamide 1 g/m², CCG-3891, Methotrexate (3 or 5 g/m²), VAC/IE, IAP, VAdC, Others | MEC | 5HT3, NK1, DEX, OLN, Other (Metoclopramide and domperidone as rescue) |
| Radhakrishnan 2024 (165) | India | LMIC | 2 arms (range): 25-70, 18-72 | 49.2 +/- 9.4 | 77.0% | Breast cancer, Head and neck cancer, Lung cancer, Others | Doxorubicin and cyclophosphamide, Cisplatin with radiation, Carboplatin-based, Cisplatin without radiation, Doxorubicin-based | HEC | 5HT3, NK1, DEX, OLN |
| Raftopoulos 2015 (166) | Multinational (United States, India, Poland) | HIC | NR | 56.6 +/- 13.1 | 74.6% | Lung, Breast, Ovarian, Lymphoma | Cyclophosphamide+anthracycline-based, Cyclophosphamide-based, Carboplatin-based, Anthracycline-based, Irinotecan-based, Carboplatin+taxane-based, Cisplatin-based, Carboplatin without taxane, Dacarbazine-based, Oxaliplatin-based, Other | HEC | 5HT3 |
| Rapoport 2010 (168) | Multinational (USA, Mexico, Canada, Chile, Brazil, Peru, Colombia, Panama, Hong Kong, Australia, South Africa, France, Germany, Israel, and Russia) | HIC | NR | 56.5 +/- 12.2 | 77.0% | Breast cancer, Colon cancer, Lung cancer, Ovarian cancer | AC (anthracycline/cyclophosphamide), non-AC. Specific agents: oxaliplatin, carboplatin, epirubicin, idarubicin, ifosfamide, irinotecan, daunorubicin, doxorubicin, cyclophosphamide, cytarabine | MEC | 5HT3, NK1, DEX |
| Rapoport 2015 (167) | Multinational (North America, Central and South America, Europe, Asia, and Africa) | HIC | Two arms (range): 59 (21-86), 59 (18-90) | NR | 37.1% | Breast, Colon or rectum, Head and neck, Lung, Ovary, Stomach, Other | Cisplatin-based highly emetogenic chemotherapy; some with concomitant taxanes | HEC | 5HT3, NK1, DEX |
| Razavi 1993(169) | Belgium | HIC | 2 arms (range): 36-66, 29-64 | 49.7 +/- 9.0 | 100.0% | Breast cancer | 4'epi-CPA (epirubicin-cyclophosphamide) high dose, 4'epi-CPA conventional dose, CMF IV (cyclophosphamide, methotrexate, and fluorouracil), CMF conventional, FEC (fluorouracil, epirubicin, and cyclophosphamide) | MEC | 5HT3, DEX, other (haloperidol, metoclopramide, alizapride, alprazolam, triazolam) |
| Roila 1991 (172) | Italy | HIC | NR | 60.0 | 43.1% | Genitourinary, Lung, Head and neck squamous, Other | CDDP (cisplatin) alone, CDDP + MTX + ADR + VLB, CDDP + CYC, CDDP + VP16, CDDP + ADR + CYC, CDDP + other | HEC | 5HT3, DEX |
| Roila 1991 (170) | NR | NR | NR | NR | NR | NR | High-dose cisplatin (CDDP) | HEC | DEX, Other (Metoclopramide) |
| Roila 2014 (171) | Italy | HIC | 50 | 53.0 | 99.8% | Breast cancer | FEC, EC, Epirubicin alone, AC | MEC | 5HT3, NK1, DEX, Other (Prochlorperazine, metoclopramide as rescue) |
| Roscoe 2010 (173) | United States | HIC | 3 arms (ranges): 28-91, 23-84, 25-90 | 53.5 +/- 11.6 | 89.3% | Alimentary Tract, Breast, Genitourinary, Gynecologic, Head and Neck, Hematologic, Lung, Other | Doxorubicin (Adriamycin), Carboplatin, Cisplatin, Cyclophosphamide | HEC | 5HT3, DEX other (prochlorperazine, acupressure bands, acustimulation band) |
| Ruff 1994 (174) | Multinational (Denmark, France, Germany, The Netherlands, South Africa, Switzerland, and the United Kingdom) | HIC | NR | 54.7 | 43.8% | Gynaecological, Lung, Head and neck, Genito-urinary, Gastrointestinal, Bone/soft tissue | MEC or HEC: Cisplatin (concomitant chemotherapy permitted: carmustine, cyclophosphamide, daunorubicin, doxorubicin, epirubicin, actinomycin D, lomustine, plicamycin, procarbazine, methotrexate, dacarbazine, mechlorethamine, streptozotocin, melphalan) | HEC | 5HT3 |
| Ruhlmann 2016 (175) | Multinational (Germany, Australia, Norway, Denmark) | HIC | Two arms (range): 48 (25-74), 47 (26-77) | NR | 100.0% | Cervical cancer | Cisplatin | HEC | 5HT3, NK1, DEX |
| Saito 2009 (176) | Japan | HIC | NR | 58.2 +/- 10.5 | 58.3% | Non-small cell lung cancer, small-cell lung cancer, breast, others | HEC: Cisplatin, or doxorubicin or epirubicin with cyclophosphamide. Concomitant agents included gemcitabine, vinorelbine and fluorouracil | HEC | 5HT3, DEX |
| Sakai 2021 (177) | Japan | HIC | 71 (34-79) | NR | 30.0% | Small cell lung cancer, Non-small cell lung cancer, Thymoma/thymic carcinoma, Others | Carboplatin (CBDCA), Pemetrexed, Bevacizumab, Pembrolizumab, Paclitaxel, Atezorizumab, Nab-paclitaxel, Etoposide, Vinorelbine, Tegafur/gimeracil/oteracil | MEC | 5HT3, DEX, OLN |
| Sandhya 2023 (178) | India | LMIC | Two arms (range): 55 (24-74), 55 (18-78) | NR | 36.3% | Gastric, Lung, Hepatopancreaticobiliary (Hepatocellular carcinoma, Periampullary carcinoma, Cholangiocarcinoma, Pancreatic, Gallbladder) | CAPOX (capecitabine/oxaliplatin), FLOT(5FU/oxaliplatin/docetaxel), DOX (docetaxel/oxaliplatin/capecitabine), GEMOX/GemCap (gemcitabine/oxaliplatin/capecitabine), Paclitaxel, Carboplatin, Pemetrexed, Cisplatin | HEC | 5HT3, NK1, DEX, OLN |
| Sato 2016 (179) | Japan | HIC | NR | 49.7 +/- 13 | 100.0% | Breast cancer | Anthracycline containing with FEC (Epirubicin hydrochloride, Cyclophosphamide, 5-FU) or EC (Epirubicin hydrochloride, Cyclophosphamide) | MEC | 5HT3, NK1, DEX, OLN |
| Schmitt 2014 (180) | Germany | HIC | 2 arms (range): 58.3 (27-72), 57.9 (35-72) | NR | 36.5% | Multiple myeloma | Melphalan | Minimal | 5HT3, NK1, DEX |
| Schnadig 2016 (181) + Schwartzberg 2016 (184) | United States | HIC | NR | 55.6 +/- 11.8 | 81.0% | NR | HEC: Anthracycline, Cyclophosphamide, Cisplatin | HEC | 5HT3, NK1, DEX |
| Schwartzberg 2015 (185) + Hesketh 2016 (94) | Multinational (North America, Central and South America, Europe, Asia, and Africa) | HIC | Two arms (range): 58 (22-86), 56 (22-88) | NR | 80.1% | Breast, Colon or rectum, Head and neck, Lung, Ovary, Stomach, Other | Cyclophosphamide, Doxorubicin, Epirubicin, Daunorubicin, Carboplatin, Irinotecan, Pemetrexed, Oxaliplatin, Ifosfamide, Cisplatin, Mitoxantrone, Idarubicin, Cytarabine | HEC | NK1, 5HT3, DEX |
| Schwartzberg 2018 (183) | Multinational (Austria, Croatia, Czech Republic, Germany, Israel, Italy, Poland, Serbia, Spain, Ukraine, and United States) | HIC | NR | 59.5 | 47.0% | Lung, Ovarian, Gastric, Head & Neck | Cisplatin, Cyclophosphamide, Dacarbazine, Carmustine, Mechlorethamine | HEC | NK1, 5HT3, DEX, other (metoclopramide) |
| Schwartzberg 2020 (182) | Multinational(U.S., Russia, Ukraine, and Georgia) | HIC | NR | 55.4 +/- 9.8 | 100.0% | Breast cancer | Anthracycline-cyclophosphamide (AC) regimens (Epirubicin + cyclophosphamide or Doxorubicin + cyclophosphamide) | MEC | NK1, 5HT3, DEX |
| Seol 2016 (186) | Korea | HIC | NR | 59.7 +/- 10.3 | 37.8% | NR | MEC, combining 5-fluorouracil/leucovorin with irinotecan (FOLFIRI) or oxaliplatin (FOLFOX) | MEC | 5HT3, DEX, other (GTDS) |
| Sledge 1992 (188) | United States | HIC | 2 arms (ranges): 29 (16-44), 28 (16-37) | NR | 0.0% | Testicular cancer, adenocarcinoma of the lung | Cisplatin + Etoposide/Bleomycin, Cisplatin + Etoposide, Cisplatin + Etoposide/Ifosfamide | HEC | 5HT3, other (metoclopramide) |
| Soukop 1990 (189) | Multinational (Belgium, France, Holland, South Africa, Switzerland, and the U.K) | HIC | NR | 53.0 | 47.6% | NR | High-dose cisplatin | HEC | 5HT3 |
| Steele 1980 (190) | United States | HIC | NR | NR | NR | NR | High-dose Cisplatin (DDP), low-dose DDP, Mechlorethamine, Actinomycin D, and DTIC. Quote: "high-dose cis-diamminedichloroplatinum(II) (DDP), low-dose DDP, mechlorethamine, actinomycin D, and dimethyltriazenoimidazolecarboxamide | HEC | Other (nabilone, prochlorperazine) |
| Stewart 1995 (191) | Multinational (Denmark, France, Germany, Holland, UK, and South Africa) | HIC | NR | 50.3 | 100.0% | Breast cancer | Cyclophosphamide-based regimens (CMF, AC, EC) | MEC | 5HT3, other (metoclopramide as a rescue) |
| Stiff 2013 (192) | United States | HIC | 2 arms (range): 51(19-79), 50 (20-75) | NR | 36.3% | Non-Hodgkin lymphoma, AML, Multiple myeloma, ALL, Hodgkin's lymphoma, CML, and Other (myelodysplastic syndrome, myeloproliferative disorder, chronic lymphocytic leukemia, myelofibrosis | Myeloablative high-dose cyclophosphamide preparative regimens (TBI/Cy, Busulfan/Cy, TBI/VP (etoposide)/Cy, BCV (BCNU, cyclophosphamide, VP)) | MEC | NK1, 5HT3, DEX |
| Strum 1984 (194) | United States | HIC | 54(Range: 22-85) | 59.6 | 56.0% | Lung, breast, malignant lymphoma, gastrointestinal neoplasms, prostate, malignant melanoma, soft tissue sarcoma, glioblastoma, leukemia, esophagus, pancreas, ovary, gallbladder, head and neck, other | HEC: Combinations of agents including nitrogen mustard, cyclophosphamide, dactinomycin, dacarbazine, 6-thioguanine, carmustine, or lomustine . MEC: Combinations of agents including cyclophosphamide, doxorubicin, carmustin, or lomustine. LEC: Combinations including methotrexate, 5-fluorouracil, vinblastine, cyclophosphamide, or doxorubicin. | HEC | Other (metoclopramide) |
| Strum 1985 (193) | United States | HIC | 2 arms: 66 and 61.5 median Ranges: (43-75, 46-78) | 62.5 | 53.3% | Lung, Head and neck, Esophagus, Ovary, and Other | Cisplatin in combination with vinblastine, etoposide (VP-16), bleomycin, or 5-fluorouracil | HEC | DEX, Other (Metoclopramide) |
| Sugawara 2019 (195) | Japan | HIC | 3 arms (range): 67.0 (36-79), 66.0(41-76), 67.0 (37-78) | NR | 24.5% | Lung and other | Cisplatin plus vinorelbine tartrate, Cisplatin plus pemetrexed, Cisplatin plus pemetrexed plus bevacizumab, Cisplatin plus etoposide, Cisplatin plus other cancer treatment | HEC | NK1, 5HT3, DEX |
| Sugimori 2017 (196) | Japan | HIC | 58.1 (37-78) | NR | 100.0% | Ovary, Uterus, Cervix, Double cancer | Paclitaxel and carboplatin combination (TC) | MEC | NK1, 5HT3, DEX |
| Sukauichai 2022 (197) | Thailand | LMIC | NR | 49.0 +/- 11.4 | 67.1% | Breast, Head-neck, Others | Doxorubicin combined with cyclophosphamide (AC) or Cisplatin | HEC | OLN, 5HT3, DEX, other (metoclopramide) |
| Sun 2005 (198) | United States | HIC | 61.3 (Range: 24.9-82.2) | 60.7 | 100.0% | Ovarian, primary peritoneal, or fallopian tube cancer | Carboplatin/paclitaxel, single-agent carboplatin, liposomal doxorubicin, single-agent paclitaxel, gemcitabine, docetaxel, carboplatin/docetaxel, topotecan | MEC | NR |
| Sun 2023 (199) | Korea | HIC | NR | 60.8 +/- 10.7 | 30.5% | Lung, Gastrointestinal, Head or neck, Breast, Other | HEC Cisplatin chemotherapy and Non-cisplatin chemotherapy | HEC | 5HT3, NK1, DEX, other (metoclopramide, lorazepam) |
| Suzuki 2016 (200) | Japan | HIC | 2 arms (range): 63 (31-77), 64 (25-83) | NR | 25.5% | Non-small-cell lung, Small-cell lung, Esophageal, Gastric, Head and neck, Other | Cisplatin HEC | HEC | 5HT3, NK1, DEX |
| Takemoto 2017 (201) + Nishimura 2015 (158) | Japan | HIC | NR | 64.4 | 39.2% | Colorectal cancer | FOLFOX, XELOX, or SOX including oxaliplatin | MEC | NK1, DEX, 5HT3 |
| Tamura 2015 (202) + Tamura 2017(203) + Iihara 2019 (97) | Japan | HIC | Range: 19-87 | 62.0 | 54.3% | Breast, Hematological malignancy, Lung, Gynecological cancer, Gastric, Colorectal cancer, Esophageal, Hepatocellular carcinoma, Biliary tract cancer, Pancreatic cancer, Other | CDDP + fluorouracil (tegafur), CDDP + gemcitabine, CDDP + fluorouracil + docetaxel, CDDP + pemetrexed, FEC, CHOP, EC, AC, TC, FOLFOX, CBDCA + pemetrexed, XELOX, and others | LEC | DEX, 5HT3, NK1 |
| Tan 2009 (204) | China | LMIC | 2 arms (ranges): 27-73, 18-74 | 52.1 +/- 11.1 | 40.2% | Lung, Stomach, Breast, Ovarian, Lymphoma, Oesophageal, Colorectal, Oropharyngeal, Teratoma, Gingival, Thymus, Cervical, Laryngeal, Malignant melanoma, Glioblastoma | MEC (oxaliplatin, carboplatin, epirubicin, adriamycin) or HEC (Cisplatin, dacarbazine) | HEC | 5HT3, DEX, OLN |
| Tanaka 2019 (205) | Japan | HIC | 75 (Range: 60-85) | NR | 12.1% | Non-small-cell lung cancer (Adenocarcinoma, Squamous cell carcinoma) and Small cell lung cancer | pemetrexed and carboplatin, or paclitaxel, and carboplatin, or S-1, and carboplatin, etoposide, and carboplatin, irinotecan | MEC | 5HT3, NK1, DEX, OLN |
| Tanioka 2013 (206) | Japan | HIC | 2 arms (range): 58 (36-67), 59 (33-69) | NR | 100.0% | Ovarian cancer, Endometrial cancer, Cervical cancer, Colorectal cancer, Non-small cell lung cancer, Cancer of primary unknow | CBDCA (AUC 6) + PTX, CBDCA (AUC 5) + PTX, CBDCA (AUC 5) + pemextrexed + bevacizumab, CBDCA (AUC 5) + PLD, FOLFIRI | Minimal | 5HT3, NK1, DEX |
| Tienchaiananda 2019 (207) | Thailand | LMIC | NR | 48.4 | 100.0% | Breast cancer (ductal and lobular) | Doxorubicin and cyclophosphamide | MEC | 5HT3, DEX, OLN, other metoclopramide |
| vanderVorst 2021 (208) | Netherlands | HIC | 65 (Range: 40-84) | NR | 44.3% | Colorectal, Ovarian, Lung, Gastric, Pancreatic, Other | Oxaliplatin-based, Carboplatin-based, Irinotecan-based, Anthracycline-based, Other | MEC | 5HT3, DEX, other (metoclopramide) |
| Vardy 2006 (209) | Canada | HIC | 53.5 | 53.0 | 77.0% | Breast cancer, Colorectal cancer, Gynaecological cancer, Lung, Prostate, Unknown | AC+/-T (Adriamycin and cyclophosphamide+/-taxane); CEF (cyclophosphamide, epirubicin, 5-fluorouracil); FOLFOX (oxaliplatin, 5-fluorouracil and folinic acid); XELIRI (capecitabine (Xeloda) and irinotecan) | MEC | 5HT3, DEX, other (prochlorperazine, domperidone, metoclopramide) |
| Vimolchalao 2020 (210) | Thailand | LMIC | 54.5 (Range: 26-73) | NR | 68.8% | Breast, Head and neck, Lung, Other | adriamycin-cyclophosphamide (adriamycin 60 mg/m2 and cyclophosphamide 600 mg/m2, AC) or high-dose cisplatin (> 70 mg/m2) regimens | HEC | 5HT3, DEX, OLN |
| Wang 2021 (211) | China | LMIC | NR | 40.1 +/- 7.3 | 100.0% | Stomach, Small intestine, Colorectum | FOLFOX (fluorouracil, leucovorin, and oxaliplatin) and FOLFIRI (fluorouracil, leucovorin, and irinotecan) | MEC | 5HT3, NK1, DEX |
| Warr 2005 (212) + Warr 2011 (213) | Multinational (United States, Germany, Austria, Canada, Hong Kong, Hungary, Spain, United Kingdom, Italy, Australia, and Greece) | HIC | NR | 52.6 +/- 10.8 | 99.8% | Breast cancer (ductal and lobular carcinoma) | Cyclophosphamide + doxorubicin, Cyclophosphamide + doxorubicin + docetaxel, Cyclophosphamide + doxorubicin + fluorouracil, Cyclophosphamide + doxorubicin + paclitaxel, Cyclophosphamide + epirubicin, Cyclophosphamide + epirubicin + fluorouracil, Cyclophosphamide + fluorouracil + methotrexate | MEC | 5HT3, NK1, DEX, other (rescue therapy: phenothiazines, butyrophenones, benzodiazepines, benzamides, domperidone) |
| Weinstein 2016 (214) | Multinational | HIC | NR | 59.6 +/- 12.1 | 59.1% | Lung, Breast, Colorectal, Gynecologic, Gastrointestinal, Head and neck, Other | Non-anthracycline and cyclophosphamide (AC)-based moderately emetogenic chemotherapy (including carboplatin, oxaliplatin) | MEC | NK1, 5HT3, DEX |
| Yahata 2016 (215) | Japan | HIC | NR | 59.0 | 100.0% | Ovarian cancer, Endometrial cancer, Cervical cancer, Peritoneal cancer, Tubal cancer | Paclitaxel and carboplatin (TC) | MEC | NK1, 5HT3, DEX |
| Yanai 2018 (216) | Japan | HIC | 2 arms (ranges): 62(36-74), 64(41-75) | NR | 29.4% | Non-small cell lung cancer, Esophageal cancer, Gastric cancer, Head and neck cancer, Small cell lung cancer, Other | Highly emetogenic chemotherapy with cisplatin | HEC | OLN, NK1, 5HT3, DEX |
| Yang 2017 (217) | China | LMIC | 2 arms (ranges): 55 (20-79), 53(18-74) | NR | 49.5% | NR | Highly emetogenic chemotherapy (primarily cisplatin-based regimens, but also AP, CAP, NP) | HEC | NK1, 5HT3, DEX |
| Ye 2022 (218) | China | LMIC | 57 (Range: 20-75) | NR | 42.0% | Non-Hodgkin lymphoma, Hodgkin lymphoma, Multiple myeloma | SEAM or high-dose melphalan | Minimal | NK1, DEX, 5HT3, OLN |
| Yeo 2020 (219) | Hong Kong | HIC | 55 years | NR | 100.0% | Breast (Ductal, lobular, other) | Doxorubicin/Cyclophosphamide (AC) | MEC | 5HT3, NK1, DEX, OLN |
| Zhang 2017 (221) | China | LMIC | 54 (Range: 22-73) | NR | 69.4% | Lung, breast, other | Cisplatin (CDDP) or Doxorubicin/Epirubicin plus Cyclophosphamide | HEC | 5HT3, DEX, other (thalidomide) |
| Zhang 2018 (220) | Multinational (China, Taiwan, Thailand, Korea) | HIC | NR | 54.6 +/- 9.9 | 29.0% | Lung, head and neck | Cisplatin-based (monotherapy or combined with Gemcitabine, Pemetrexed, Docetaxel, Etoposide, or Fluorouracil) | HEC | NK1, 5HT3, DEX |
| Zhao 2023 (223) | China | LMIC | 2 arms (range): 59(21-82), 59 (24-80) | NR | 25.8% | Lungs, Urogenital, Digestive, Others | Cisplatin containing regimen | HEC | NK1, 5HT3, DEX |
| Zhao 2023 (222) | China | LMIC | 2 arms(range): 60 (20-73), 58 (20-77) | NR | 22.3% | Lung, head and neck, other | Multiday cisplatin-based chemotherapy | HEC | OLN, NK1, 5HT3, DEX |
